# Prevention of Bacterial Contamination of a Silica Matrix Containing Entrapped β-Galactosidase through the Action of Covalently Bound Lysozymes

**DOI:** 10.3390/molecules22030377

**Published:** 2017-02-28

**Authors:** Heng Li, Shuai Li, Pu Tian, Zhuofu Wu, Zhengqiang Li

**Affiliations:** 1 Key Laboratory for Molecular Enzymology and Engineering of the Ministry of Education, College of Life Sciences, Jilin University, Changchun 130012, China; liheng12@mails.jlu.edu.cn (H.L.); ls2012@jlu.edu.cn (S.L.); tianpu@jlu.edu.cn (P.T.); 2 Informalization Center for Education and Management, Jilin Agricultural University, Changchun 130118, China; 3 College of Life Science, Jilin Agricultural University, Changchun 130118, China

**Keywords:** β-galactosidase, lysozyme, encapsulation, covalent binding

## Abstract

β-galactosidase was successfully encapsulated within an amino-functionalised silica matrix using a “fish-in-net” approach and molecular imprinting technique followed by covalent binding of lysozyme via a glutaraldehyde-based method. Transmission electron microscopy (TEM), X-ray diffraction (XRD), scanning electron microscopy (SEM), and Fourier transform infrared (FTIR) spectroscopy were used to characterise the silica matrix hosting the two enzymes. Both encapsulated β-galactosidase and bound lysozyme exhibited high enzymatic activities and outstanding operational stability in model reactions. Moreover, enzyme activities of the co-immobilised enzymes did not obviously change relative to enzymes immobilised separately. In antibacterial tests, bound lysozyme exhibited 95.5% and 89.6% growth inhibition of *Staphylococcus aureus* ATCC (American type culture collection) 653 and *Escherichia coli* ATCC 1122, respectively. In milk treated with co-immobilised enzymes, favourable results were obtained regarding reduction of cell viability and high lactose hydrolysis rate. In addition, when both co-immobilised enzymes were employed to treat milk, high operational and storage stabilities were observed. The results demonstrate that the use of co-immobilised enzymes holds promise as an industrial strategy for producing low lactose milk to benefit people with lactose intolerance.

## 1. Introduction

In industrial applications, β-galactosidase is used to convert lactose to glucose and galactose, producing low lactose milk for individuals suffering from lactose intolerance [1]. However, the cost of β-galactosidase and its sensitivity to environmental changes have limited its utilisation [2]. Immobilisation techniques have been shown to enable easier separation of enzymes from reaction media to facilitate enzyme reuse, while also enhancing enzyme performance by improving enzyme stability, activity, specificity, and selectivity and reducing inhibition [3,4,5,6,7]. For instance, the stabilisation of monomeric enzymes can be improved by multipoint covalent attachment or generation of favourable environments surrounding enzymes [8], while the stabilisation of multimeric enzymes can be boosted by multi-subunit covalent immobilisation to avoid subunit dissociation [9]. Moreover, immobilisation may improve enzyme activity through inhibition of aggregation, while concurrently bolstering enzyme structure to preserve enzymatic activity, even under drastic conditions [10]. When inhibition of enzyme activity results from a high concentration of substrate, product, or other components in the reaction medium, such inhibition may be diminished by deliberate conformational distortion of enzymes through the use of immobilisation strategies [10,11,12]. Furthermore, the specificity and selectivity of immobilised enzymes can be markedly elevated through rational distortion of their active sites, induction of enzyme conformational changes, or by altering the interaction between an enzyme and its carrier substrate [7,10,11,13]. In our previous work, β-galactosidase was successfully incorporated into a silica matrix using a “fish-in-net” encapsulation approach and molecular imprinting technique; the encapsulated β-galactosidase exhibited an increase in specific activity of 145% compared with free enzyme [14]. Unfortunately, β-galactosidase incorporated within a silica matrix is vulnerable to bacterial contamination during continuous production of low lactose milk [15], and this issue must be addressed.

In recent years, many chemical agents, such as organic and inorganic acids, alcohols, ammonium compounds and amines, as well as metal ion leachates mainly from silver and copper, have been used to reduce bacterial contamination [16,17,18,19,20,21]. However, most chemical antibacterial agents exhibit toxicity and short-term antimicrobial efficacy [21,22,23]. Therefore, growing attention is currently focused on natural antimicrobial agents, which are recognised by the food industry as safe alternatives [24]. Among natural antimicrobial agents, lysozyme is the most noteworthy antibacterial substance [25]. Lysozyme, an effective antifungal and antibacterial agent, damages bacterial cell walls by catalysing hydrolysis of murein β-1,4-glycosidic bonds to prevent bacterial adhesion and biofilm formation [26,27]. In this study, lysozyme was affixed to the surface of a host silica matrix encapsulating β-galactosidase to prevent bacterial growth during milk production.

In general, co-immobilisation of enzymes is necessary for one-pot cascade synthesis reactions [28,29]. Furthermore, co-immobilisation of enzymes is advantageous for performance of domino reactions, enabling the second enzyme in a sequence to receive a higher concentration of substrate from the very beginning of the reaction run [30,31]. Moreover, co-immobilisation of enzymes is useful in cases where the activity of the first enzyme is damaged by its product or its product is unstable [32,33]. Nevertheless, several problems may hinder utilisation of co-immobilisation methods. First, enzymes co-immobilised onto a common carrier lose their usefulness once the least stable enzyme loses its activity. Second, a single protocol to co-immobilise all one-pot cascade enzymes to a common support may not be optimal for each enzyme [11,34,35]. Therefore, in this work, lysozyme and β-galactosidase immobilisations were accomplished separately to preserve maximal activity for each enzyme.

Ensuring that an enzyme remains tightly fixed to a carrier via covalent bonds may be the best strategy to prevent enzyme leaching and protein contamination of the product [36,37,38,39]. Because glutaraldehyde is one of the simplest and most gentle coupling reagents to covalently bond an enzyme to a substrate [40,41,42], it was selected in this work to covalently affix lysozyme to the surface of the host silica matrix. In general, glutaraldehyde molecules bound to the ε-amino groups of the lysine residues of an enzyme can covalently react with the primary amino groups of the support to establish a multi-point covalent enzyme-support attachment [43]. For this reason, amino groups were incorporated into the silica matrix using tetraethoxysilane (TEOS) and 3-aminopropyltriethoxysilane (APTES).

**Figure 1:**
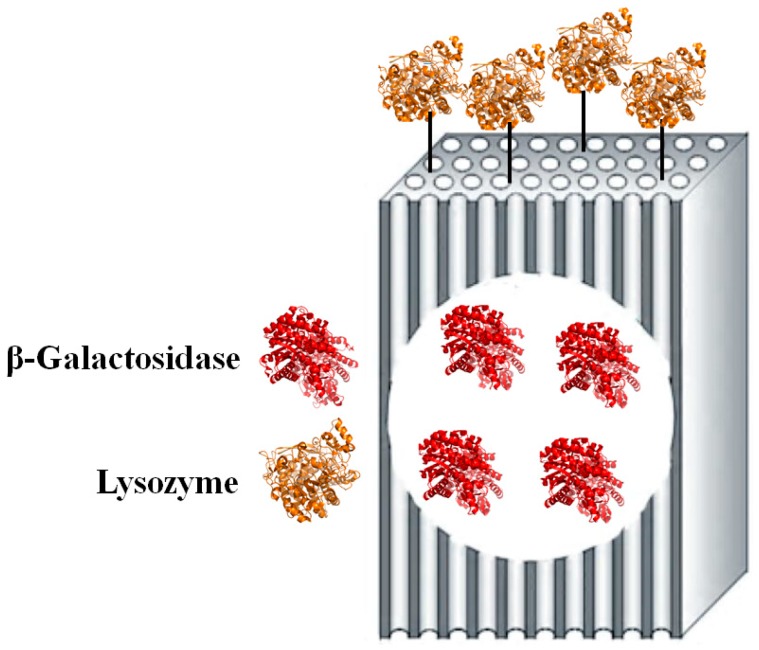
The strategy for co-immobilisation of the two enzymes relative to the silica substrate.

In this work, co-immobilisation of enzymes was achieved using a two-step procedure (Figure 1). First, β-galactosidase was encapsulated within the amino-functionalised silica matrix using the “fish-in-net” approach. Second, lysozyme was covalently bound to the surface of the same support using glutaraldehyde as a crosslinking agent. After covalent bonding of lysozyme, the silica matrix with co-immobilised enzymes was characterised using transmission electron microscopy (TEM), X-ray diffraction (XRD), scanning electron microscopy (SEM), and Fourier transform infrared spectroscopy (FTIR). Next, the specific activities of both enzymes co-immobilised onto the same support vs. individually immobilised enzymes were compared. In addition, the reusability of the encapsulated β-galactosidase and bound lysozyme were evaluated in model reactions, while in vitro antimicrobial activity of bound lysozyme toward *Staphylococcus aureus* ATCC (American type culture collection) 653 and *Escherichia coli* ATCC 1122 was also assessed. Finally, to test the practical value of the strategy evaluated in this work, assessment of lactose hydrolysis in milk after treatment using co-immobilised enzymes was performed, and assessments of co-immobilised enzyme stabilities during milk treatment, reuse, and prolonged storage were conducted.

## 2. Results and Discussion

### 2.1. Sample Characterization by TEM and XRD

**Figure 2:**
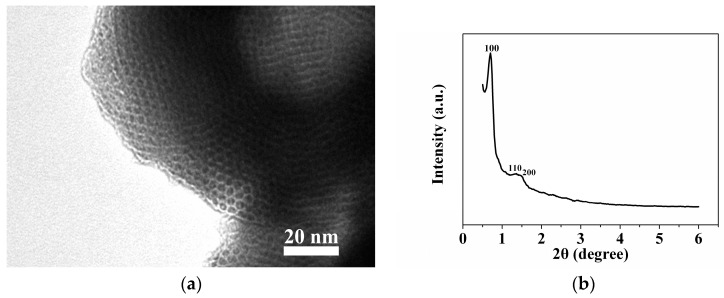
Transmission electron microscope (TEM) image (**a**) and X-ray pattern (**b**) of the silica matrix containing both enzymes.

As can be seen from Figure 2a, the TEM image of co-immobilised enzymes demonstrates well-ordered hexagonal arrays of mesoporous channels (1D channels). The small-angle X-ray pattern of co-immobilised enzymes shows three peaks indexed to (100), (110), and (200) reflections corresponding to a two-dimensional hexagonal *P6mm* structure with a large unit–cell parameter (Figure 2b). Cheng and colleagues have reported that well-ordered mesoporous materials with amino functionality can be obtained using a co-condensation method involving TEOS and APTES with the triblock copolymer Pluronic P123 (EO_20_PO_70_EO_20_) as a mesopore structure-directing agent [44], which is in agreement with our results. Moreover, our results confirm that the presence of β-galactosidase does not perturb the mesostructure of the co-immobilised enzymes when using TEOS and APTES as silica amino group sources.

### 2.2. Sample Characterization by SEM 

**Figure 3:**
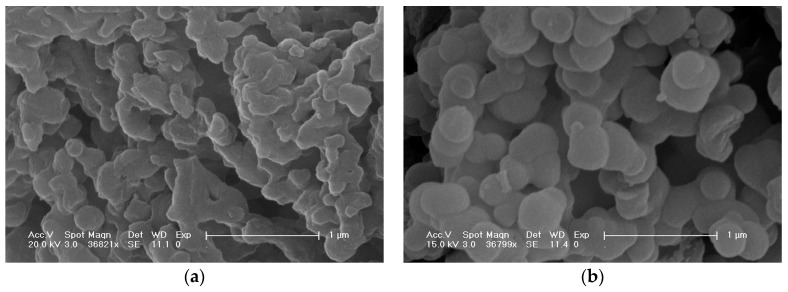
Scanning electron microscope (SEM) images of NH_2_-Silica (**a**) and co-immobilised enzymes (**b**).

The SEM image of NH_2_-Silica exhibits irregular fragments, while co-immobilised enzymes possess spherical morphologies with particle sizes of 200–500 nm (Figure 3). The surfaces of the matrix with co-immobilised enzymes are smooth (Figure 3b) as compared with NH_2_-Silica (Figure 3a). It is well known that certain biomolecules such as proteins, enzymes, or antibodies can direct the polymerisation of silica and silicones in vitro or in vivo [45,46,47,48]. Hence, we speculate that β-galactosidase may act as a bio-template to direct morphological development of the amino-functionalised silica matrix as it undergoes hydrolysis and silicate condensation during synthesis.

### 2.3. Sample Characterization by FTIR Spectra

**Figure 4:**
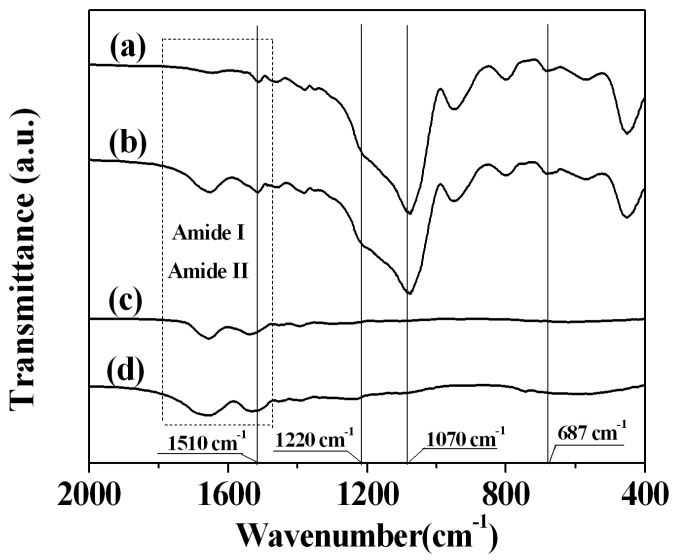
Fourier transform infrared (FTIR) spectrum of NH_2_-Silica (**a**); the co-immobilised enzymes (**b**); lysozyme (**c**); and β-galactosidase (**d**).

To confirm the presence of both enzymes in the matrix, the FTIR spectra of the amino-functionalised silica matrix in the presence or absence of lysozyme and β-galactosidase, lysozyme and β-galactosidase were surveyed. Typical Si-O-Si bands can be observed at 1220 cm^−1^ and 1070 cm^−1^ in the spectrum (Figure 4, curve a), which demonstrates the formation of a condensed silica network [49,50]. The amide I and II bands of lysozyme (Figure 4, curve c) and β-galactosidase (Figure 4, curve d) can be observed for separate enzymes. After co-immobilisation, characteristic bands for amide I and amide II of both enzymes and Si–O–Si can be observed (Figure 4, curve b), which confirms that lysozyme and β-galactosidase were successfully immobilised to the amino-functionalised silica matrix. Moreover, the absorbance peaks at 1510 cm^−1^ and 687 cm^−1^ (curve a) are attributed to the N–H bending vibration and the symmetric NH_2_ bending vibration, which verifies the incorporation of the amino group in the silica matrix (Figure 4) [44]. As for curve b, the absorbance peaks at 1510 cm^−1^ and 687 cm^−1^ are ascribed to both the amino groups incorporated in the silica matrix framework and also to the amino groups of the enzymes (Figure 4).

### 2.4. The Activities of the Co-Immobilised Enzymes

The experimental results verify that bound lysozyme exhibits 96.3% of antimicrobial activity as compared with free lysozyme (Table 1). In this experiment, the pore of the amino-functionalised silica matrix was blocked by triblock copolymer P123 during the covalent binding of lysozyme; in this way, the lysozyme was covalently grafted mainly to the surface of the amino-functionalised silica matrix rather than within matrix channels. This is important because lysozyme bound to the surface of the matrix is more apt to contact and degrade bacterial cell walls to effect subsequent cell lysis. The slight loss of antimicrobial activity may be due to the interaction between lysozyme and the amino-functionalised silica matrix, causing a change in lysozyme conformation [51]. The hydrolytic activity of the encapsulated β-galactosidase did not change before or after the covalent binding of lysozyme (Table 1), suggesting that the lysozyme bound to the surface of the amino-functionalised silica matrix did not disturb the diffusion of substrates and products during lactose hydrolysis. The experimental data also verify that the entrapment of β-galactosidase does not influence the antimicrobial activities of lysozyme (Table 1). Therefore, the preservation of antimicrobial activity shall be attributed to the fact that each enzyme was immobilised separately on the host silica matrix using an immobilisation strategy that did not adversely affect either enzyme, in accordance with previously published reports [34,35].

### 2.5. The Operational Stability of the Co-Immobilised Enzymes

In practical applications, the reusability of enzymes is of considerable importance and enzyme immobilisation can facilitate enzyme recovery [52,53,54,55]. To assess enzyme reusability, the silica matrix with co-immobilised enzymes was evaluated over multiple cycles for bactericidal activity. The results demonstrate retention of 96.6% of initial matrix antimicrobial activity even after 10 runs (Table 2), with negligible loss of antimicrobial activity observed throughout the experiment. The results indicate that lysozyme firmly binds to the surface of the amino-functionalised silica matrix through multipoint covalent attachments between the enzyme and the support [7]. Furthermore, after ten cycles, the encapsulated β-galactosidase still retained 99.8% of its initial hydrolytic activity (Table 2).

### 2.6. Antibacterial Activity Assessments

**Figure 5:**
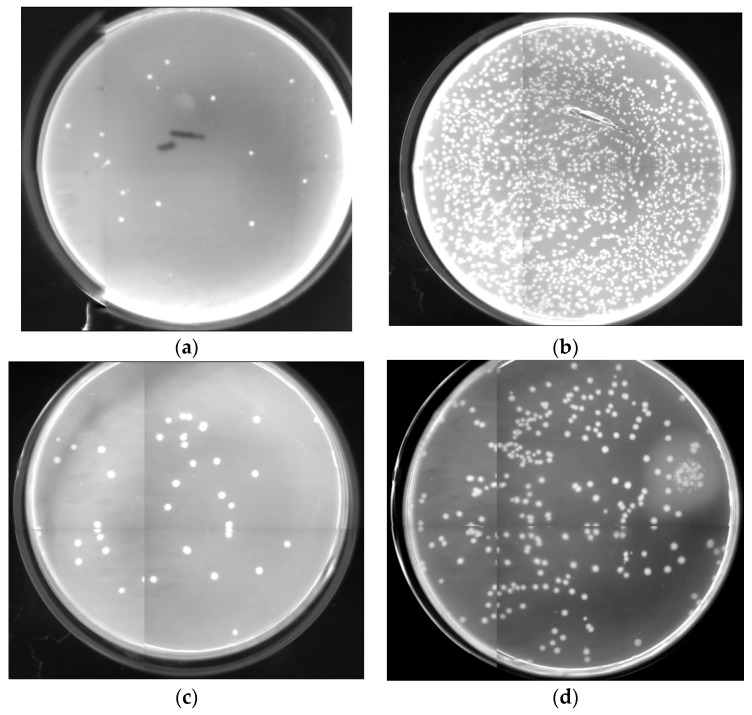
Antibacterial assay on Petri dishes: *Staphylococcus aureus* ATCC (American type culture collection) 653 incubated with the co-immobilised enzymes (**a**); *Staphylococcus aureus* ATCC 653 incubated with NH_2_-Silica (**b**); *Escherichia coli* ATCC 1122 incubated with the co-immobilised enzymes (**c**); *Escherichia coli* ATCC 1122 incubated with NH_2_-Silica (**d**).

The experimental data demonstrate the presence of a conspicuous antimicrobial activity of bound lysozyme in vitro. As shown in Figure 5, this activity was tested using *Staphylococcus aureus* ATCC 653 and *Escherichia coli* ATCC 1122. The degree of inhibition produced by the co-immobilised enzymes and NH_2_-Silica for each organism was quantified using a colony plate count assay. Growth of *Staphylococcus aureus* ATCC 653 exhibited 95.5% inhibition, while *Escherichia coli* ATCC 1122 exhibited 89.6% growth inhibition. In general, lysozyme can lyse Gram-positive bacteria by disrupting their cell wall structures, whereas the binding of lysozyme to Gram-negative bacteria can be precluded by the outer membranes of these organisms [56]. Thus, the antibacterial activity of immobilised lysozyme toward Gram-positive *Staphylococcus aureus* ATCC 653 was higher (Figure 5a) than for Gram-negative *Escherichia coli* ATCC 1122 (Figure 5c) relative to respective silica matrix controls (Figure 5b,d).

### 2.7. The Properties of the Co-Immobilised Enzymes in Milk

An in vitro antimicrobial assay was performed to determine the antimicrobial properties of co-immobilised enzymes in milk. After 8 h of treatment, the number of viable cells decreased by 89.4% (see Supplementary Materials, Figure S1a). Meanwhile, high lactose hydrolysis (88.9%) was achieved that approached an appropriate lactose hydrolysis level for industrial low lactose milk production (see Supplementary Materials, Figure S1b) [57,58]. Moreover, during ten consecutive cycles, the changes in relative cell viability and milk lactose hydrolysis rate were very small, implying that most of the enzymes could not escape from the silica matrix during multiple cycles of reuse. Furthermore, the weight of the silica matrix loaded with the enzymes almost remained constant throughout all ten runs, indicating that the silica matrix was nearly completely separated from the milk after each cycle. Furthermore, the hydrolytic and antimicrobial activities of the co-immobilised enzymes slightly dropped for three months, suggesting that most lysozymes and β-galactosidases remained bound to the silica matrix during prolonged storage. These results indicate that co-immobilised enzymes should be useful in industrial-scale applications for producing lactose-free milk.

## 3. Materials and Methods

### 3.1. Material

β-Galactosidase from *Aspergillus oryzae* (with activity not less than 14,000 U/g) was purchased from Amano Enzymes Co. (Nagoya, Japan). Tetraethylorthosilicate (TEOS) and Pluronic P123 (EO_20_PO_70_EO_20_, *Mav* = 5800) were purchased from Sigma-Aldrich (St. Louis, MO, USA). 3-Aminopropyltriethoxysilane (APTES) was purchased from Fluka (Buchs, Switzerland). Lysozyme was purchased from Amresco (Solon, OH, USA). Spectral-grade KBr was purchased from BDH Co., Ltd. (Poole, UK). The glucose oxidase-peroxidase kit was purchased from Beijing BHKT Clinical Reagent Co., Ltd. (Beijing, China). All other chemicals and reagents were of analytical grade. All aqueous solutions were prepared with Milli-Q water (Millipore, Billerica, MA, USA). *Micrococcus lysodeikticus* ATCC No. 4698 cells were obtained from Sigma-Aldrich. Bacterial strains *Staphylococcus aureus* ATCC 653 and *Escherichia coli* ATCC 1122 were purchased from the China General Microbiological Culture Collection Center (CGMCC, Beijing, China).

### 3.2. Modified “Fish-in-Net” Encapsulation

The encapsulation of β-galactosidase was carried out based on the “fish-in-net” approach and molecular imprinting technique with a slight modification [14]. Briefly, 25 mL of 37.5% (*w*/*v*) lactose solution was added to 150 mL β-galactosidase solution. Next, the mixture was flash-frozen at −80 °C in liquid nitrogen for 20 min and lyophilised soon after. The synthesis mixture had a molar composition of TEOS/APTES/P123/H_2_O/ethanol/HCl of 1/0.25/0.015/5.3/18.1/0.3 and a final pH value of 5.0. After removal of ethanol under vacuum for 72 h at ambient temperature, the preformed precursors were assembled in the glycerol solution at room temperature. β-galactosidase (0.6 g) pretreated with lactose was added to 20 g of the precursors at 4 °C and 5 mL of the buffer was added to this system simultaneously with magnetic stirring. The system was then incubated at 4 °C for 72 h to sufficiently form hydrogel. A small amount of sample was washed with phosphate-buffered saline (PBS) buffer at 4 °C to remove the polymer surfactant (P123) in the hydrogel and tested for successful β-galactosidase encapsulation. The remaining sample was stored at 4 °C and then employed in the covalent binding of lysozyme.

### 3.3. Glutaraldehyde Coupling Procedure

The amino-functionalised silica matrix loaded with β-galactosidase (200 mg) was added into 4 mL of glutaraldehyde (2.0% (*v*/*v*), aqueous solution) and the mixture was stirred at room temperature for two hours. After centrifugation, the resulting sample was washed several times with distilled water, centrifuged, and then the precipitate was added into 10 mL of lysozyme solution (2.0 mg/mL, prepared in PBS, pH 8.0). Next the suspension was incubated in a water bath at 4 °C for 6 h. Because the triblock copolymer P123 blocked the diffusion of free lysozyme into pore channels, the lysozyme was bound to the surface of the silica matrix rather than within the pore channels. It is known that ethanol extraction is a mild and efficient method to remove surfactants during the preparation of the mesoporous silica material [59]. After the binding of lysozyme, the abovementioned sample was washed several times with buffer containing 40% ethanol to remove the triblock copolymer P123, thus opening the mass transfer channels within the silica matrix. The final silica matrix product was stored in the same buffer. The immobilisation yields of β-galactosidase and lysozyme were measured using the Bradford method [60] and were quantified according to the difference between the total amount of enzyme added to the immobilisation system and enzyme recovered in the pooled supernatant and washing solutions. The immobilisation yields of β-galactosidase and lysozyme were 96.2% and 93.5%, respectively. The loading amounts of encapsulated β-galactosidase and bound lysozyme were 104 mg/g support and 139.6 mg/g support, respectively. The amino-functionalised silica matrix loaded with β-galactosidase and lysozyme is designated as “co-immobilised enzymes”. The amino-functionalised silica matrix without β-galactosidase and lysozyme is designated “NH_2_-Silica”.

### 3.4. Characterisation of the Matrix

Transmission electron microscopy was performed using an FEI Tecnai G2 F20 S-twin (FEI, American, Hillsboro, OR, USA) at 200 kV. Powder X-ray diffraction (PXRD) data were collected on a Rigaku D/Max 2550 diffractometer (Rigaku Co., Tokyo, Japan) with Cu Kα radiation (λ = 1.5418 Å) over the 2θ range of 4°–40° at room temperature. SEM experiments were performed using a JSM-6700F field emission scanning electron microscope (JEOL, Tokyo, Japan) with an acceleration voltage of 150 kV [44]. FTIR spectra were surveyed using a Nicolet 5700 FTIR spectrometer (Thermo Fisher Scientific, Inc., Waltham, MA, USA) with a resolution of 4 cm^−1^ using the KBr method.

### 3.5. The Catalytic Behaviour of the Co-Immobilised Enzymes

#### 3.5.1. Lactose Assay

The enzyme activity of β-galactosidase was measured using lactose as a substrate according to our previous method with a slight modification [14]. The reaction system contained 100 µL of lactose (1.0 M) and 900 µL of PBS buffer (pH 7.0, 0.05 M). The hydrolytic reaction was triggered by adding the enzyme and the reaction mixture was incubated in a water bath at 37 °C for 10 min. Thereafter, the reaction vessel was placed in boiling water for 5 min to stop the reaction, which was then followed by centrifugation at 3500× *g* for 3 min. The amount of generated glucose was determined using a glucose oxidase-peroxidase method. One unit of β-galactosidase activity was defined as the amount of β-galactosidase that liberates 1 µmol glucose per min under defined conditions.

#### 3.5.2. Antimicrobial Activity of Bound Lysozyme

After washing, the co-immobilised enzymes were suspended in buffer and allowed to stand for 10 min. Lysozyme activity was assayed using a published procedure [61]. *Micrococcus lysodeicticus* ATCC 4698 was cultured on Difco Nutrient Agar (Difco Laboratories, Detroit, AL, USA) for 48 h. The bacteria were washed with buffer to remove the agar and then were diluted with buffer to obtain an optical density of 1.3 at 450 nm. The co-immobilised enzymes (1 mL) were added to 9 mL of diluted bacterial solution at 37 °C. An aliquot of the reaction mixture (1 mL) was taken at 30 s intervals and then centrifuged at 1500× *g* for 1 min. The UV absorbance of the supernatant at 450 nm was monitored using a Shimadzu UV-2550 spectrophotometer (Kyoto, Japan). One unit of lysozyme activity was defined as the amount of lysozyme to cause a decrease in absorbance at 450 nm of 0.001 per min under these conditions.

#### 3.5.3. Antimicrobial and Lactose Hydrolysis Assays in Milk

Properly diluted co-immobilised enzyme-bound silica matrix was added to 50 mL of milk and incubated at 37 °C for ten hours. An aliquot of the reaction mixture (1 mL) was taken at each 1 h interval and aliquots were centrifuged at 1500× *g* for 1 min. Each suspension was then collected into a separate sterile tube. As for the antimicrobial assay, the serial dilutions of the suspension were incubated in Petri dishes containing agar medium with gentle stirring at 50 rpm to obtain sequestered bacterial punctiform colonies at 37 °C for 48 h. The survival ratio of cells was defined as the percentage of viable cells in suspensions relative to the total number of cells in untreated milk. As for the lactose hydrolysis assay, the amount of generated glucose in the suspension was determined using a method based on glucose oxidase–peroxidase activity. From the amount of generated glucose, residual lactose levels during hydrolysis were calculated based on the stoichiometry of the reaction.

### 3.6. Reusability

To test reusability, the co-immobilised enzymes were analysed in successive batches. The hydrolytic activity and antimicrobial activity of the co-immobilised enzymes were measured according to the assay described in Section 3.5.1 and Section 3.5.2, respectively. Relative cell viability and lactose hydrolysis rates in milk were measured as described in Section 3.5.3. At the end of each reaction cycle, the co-immobilised enzymes were collected from the reaction medium and washed with PBS buffer solution (pH 8.0) to remove any substrate or product retained in the NH_2_-Silica matrix. The co-immobilised enzymes were then used again for the subsequent reaction cycle. This procedure was repeated for ten cycles. In parallel testing, the silica matrix was collected by centrifugation after each cycle, dried overnight at 60 °C and weighed.

### 3.7. Antimicrobial Tests

Bacterial strains were routinely maintained at 4 °C on Luria–Bertani (LB) Nutrient Agar. Bacterial strains were always harvested during the exponential growth phase, i.e., after 4 h culture at 37 °C under constant rotation at 100 rpm. To test antimicrobial activity, 15 mL of bacterial suspension (10^5^ cells/mL) was added to 50 mL of LB medium (containing 10.0 g of peptone, 10.0 g of NaCl, and 15.0 g of agar per litre) followed by the addition of NH_2_-Silica or the co-immobilised enzyme matrix (20 mg). After 20 h at 37 °C, the serial dilutions of these suspensions were incubated in Petri dishes with gentle stirring at 50 rpm to obtain sequestered bacterial punctiform colonies. Percentages of inhibition were determined from the differences in colony numbers in controls and tests after 20 h incubation at 37 °C. The percentage of bacterial inhibition by bound lysozyme was inferred from the difference between the number of emerging colonies in controls and in tests. The antibacterial ratio (R) was determined using the following equation: R (%) = 100 × (A − B)/A, where A and B are the average numbers of colonies grown with NH_2_-Silica and the co-immobilised enzyme matrix, respectively.

### 3.8. The Storage Stability of the Co-Immobilised Enzymes

Storage stability of the co-immobilised enzymes was investigated by measuring their relative cell viability, lactose hydrolysis rate, and hydrolytic and antimicrobial activities after storage in buffer at 4 °C for a three-month period. The abovementioned measurements were performed once a week. The in vitro antimicrobial assay and lactose hydrolysis assay in milk were each performed after 8 h of treatment.

### 3.9. Statistical Analysis

The data expressed in various studies was plotted using SigmaPlot-9 and expressed as (±) standard error. Each value represents the mean for three independent experiments performed in duplicate, with average standard deviations <5%.

## 4. Conclusions

In order to produce lactose-free milk, cost-effective and efficient strategies achieving both high lactose hydrolysis rates with low bacterial growth are sought. In this study, lysozyme was covalently immobilised using glutaraldehyde crosslinking to the surface of a β-galactosidase-loaded amino-functionalised silica matrix. The amino-functionalised silica matrix containing both enzymes possessed a two-dimensional hexagonal *P6mm* structure with a large unit–cell parameter, which is beneficial for the diffusion of substrates and products. In addition, β-galactosidase in the silica matrix showed excellent hydrolytic activity and operational stability after covalent binding of lysozyme. Concurrently, the lysozyme bound to the surface of the amino-functionalised silica matrix exhibited high antibacterial activity and good operational stability. Furthermore, neither enzyme co-immobilised onto the host silica matrix lost enzymatic activity relative to matrix samples with separately immobilised enzymes. Additionally, amino-functionalised silica matrix loaded with both enzymes exhibited a high rate of growth inhibition of *Staphylococcus aureus* ATCC 653 and *Escherichia coli* ATCC 1122. When used to treat milk, co-immobilised enzymes exhibited dramatic reduction in bacterial cell viability and a high lactose hydrolysis rate, as well as excellent storage and operational stability during reuse in actual low lactose milk production. Therefore, these results pave the way for production of low lactose milk using amino-functionalised silica matrix loaded with β-galactosidase and lysozyme. Of special note, this immobilisation strategy should also be more generally applicable to combi-biocatalysts as well as other enzymes that could be employed in food and medicine production. Further investigations of such applications are currently underway in our laboratory.

## Figures and Tables

**Table 1:** The comparison of the specific activities of free and immobilised enzymes.

Sample	Specific Activity (U/mg)
Lysozyme	
Free lysozyme	10784
Immobilised lysozyme without β-galactosidase	10380
Immobilised lysozyme entrapped by β-galactosidase	10385
β-galactosidase	
Free β-galactosidase	390
Encapsulated β-galactosidase before covalent binding of lysozyme	566
Encapsulated β-galactosidase after covalent binding of lysozyme	563

**Table 2:** Antimicrobial activity and lactose hydrolytic activity of the co-immobilised enzymes during continuous operation.

Batch	Immobilised Lysozyme		Encapsulated β-Galactosidase	
	Specific Activity (U/g Support)	Residual Activity (%)	Specific Activity (U/g Support )	Residual Activity (%)
1	1.45 × 10^6^	100.0%	58,864.0	100.0%
2	1.45 × 10^6^	100.0%	58,864.0	100.0%
3	1.44 × 10^6^	99.3%	58,864.0	100.0%
4	1.44 × 10^6^	99.3%	58,864.0	100.0%
5	1.43 × 10^6^	98.6%	58,864.0	100.0%
6	1.43 × 10^6^	98.6%	58,801.6	99.9%
7	1.42 × 10^6^	97.9%	58,801.6	99.9%
8	1.41 × 10^6^	97.2%	58,801.6	99.9%
9	1.41 × 10^6^	97.2%	58,749.6	99.8%
10	1.40 × 10^6^	96.6%	58,749.6	99.8%

## Supplementary Materials

Supplementary Materials are available online.
