# Supplementary material for: Prevention of Bacterial Contamination of a Silica Matrix Containing Entrapped β-Galactosidase through the Action of Covalently Bound Lysozymes
Source: Molecules. 2017 Feb 28;22(3):377. doi: 10.3390/molecules22030377 (PMC6155228; doi:10.3390/molecules22030377)
Supplement: Supplementary file 1 [file molecules-22-00377-s001.pdf]

Supporting information

Prevention of bacterial contamination of a silica matrix containing entrapped β-galactosidase through the action of covalently bound lysozyme

Heng Li [1,2], Shuai Li [1], Pu Tian [1], Zhuofu Wu [3,*] and Zhengqiang Li [1,*]

[1] Key Laboratory for Molecular Enzymology and Engineering of the Ministry of Education, College of Life Sciences, Jilin University, Changchun 130012, China; liheng12@mails.jlu.edu.cn (H.L.); ls2012@jlu.edu.cn (S.L.); tianpu@jlu.edu.cn (P.T.)
[2] Informatization center for education and management, Jilin Agricultural University, Changchun 130118, China
[3] College of Life Science, Jilin Agricultural University, Changchun 130118, China
* Correspondence: wzf@jlau.edu.cn (Z.W.); lzq@jlu.edu.cn (Z.L.); Tel.: +86-431-8515-5201 (Z.L.)

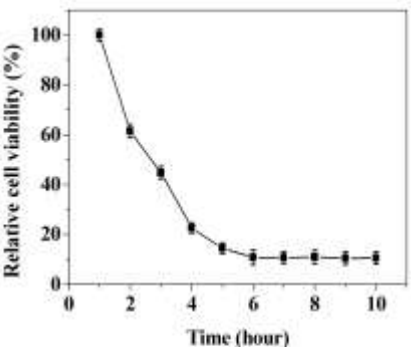

(a)

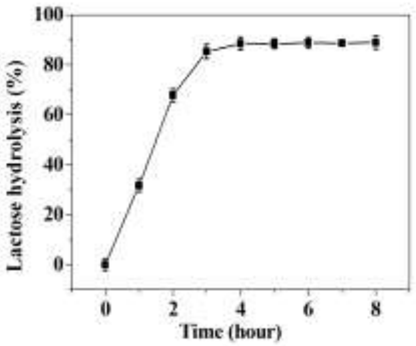

(b)

Figure S1. Time course of microbial inactivation (a) and lactose hydrolysis (b) in milk treated with co-immobilised enzymes.
